# Myopic foveal detachment associated with pachychoroid characteristics

**DOI:** 10.1186/s12886-021-02040-z

**Published:** 2021-07-28

**Authors:** Yong Kyun Shin, Sun Hyup Han, Se Woong Kang, Sang Jin Kim, A Young Kim

**Affiliations:** Department of Ophthalmology, Samsung Medical Center, Sungkyunkwan University School of Medicine, #81 Irwon-ro, Gangnam-gu, Seoul, 06351 South Korea

**Keywords:** Myopic nontractional foveal detachment, Myopia, Central serous chorioretinopathy, Pachychoroid neovascularization

## Abstract

**Purpose:**

To describe myopic nontractional foveal detachment associated with pachychoroid diseases.

**Methods:**

This retrospective study included 15 myopic eyes which had nontractional serous foveal detachment. The eyes were divided into myopic central serous chorioretinopathy (CSC) group (*n* = 8) and a myopic pachychoroid neovascularization (PNV) group (*n* = 7) according to the presence of type 1 choroidal neovascularization on multimodal imaging. The findings of multimodal imaging and treatment response were described.

**Results:**

In myopic CSC group, pachychoroid features such as pachyvessels, choroidal vascular hyperpermeability and punctate hyperfluorescent spots were noted in 8 eyes (100%), 8 eyes (100%), 5 eyes (62.5%) respectively. The above features were noted in 7 eyes (100%), 5 eyes (83.3%), 5 eyes (83.3%), respectively, in the myopic PNV group. Five of 8 eyes in myopic CSC and all 7 eyes received treatment including anti-vascular endothelial growth factor injection and/or photodynamic therapy. However, only five eyes had a complete response.

**Conclusions:**

The pachychoroid phenotype may coexist with high myopia and lead to myopic nontractional serous foveal detachment. Our series suggest that the response to treatment for these conditions would be limited.

## Summary statement

Pachychoroid phenotypes were identified in myopic eyes with nontractional serous foveal detachment and were divided two groups: myopic central serous chorioretinopathy and myopic pachychoroid neovasculopathy.

## Background

High myopia has been increasing globally and has become one of the major causes of visual impairment, especially in Asia [1–3]. Among various complications associated with high myopia, foveal detachment is one of the clinical manifestations that inevitably lead to visual impairment. This finding is usually observed in myopic traction maculopathy, macular hole, or myopic choroidal neovascularization (CNV) [4, 5]. high myopia is characterized by markedly thin choroid compared to the normal eye [6]. However, foveal detachment has been observed in the pachychoroid diseases. Pachychoroid diseases share common characteristics such as a thick choroid, dilated outer choroidal vessel (pachyvessel), inner choroidal attenuation, and a history of central serous chorioretinopathy (CSC); drusen, pigmentary abnormality, and geographic atrophy are absent [7]. The pathogenesis of pachychoroid diseases is unknown, but pachychoroid features, such as choroidal congestion and hyperpermeability, are generally associated with the focal disruption of retinal pigment epithelium (RPE) and Bruch’s membrane, which can lead to subretinal fluid (SRF) and CNV [8]. Although both high myopia and pachychoroid diseases may develop exudative complications, the two disease entities are perceived as incompatible due to inherent structural differences, mainly the choroidal thickness. However, high myopia has presented with serous non-tractional foveal detachments, typical clinical features of pachychoroid diseases. The purpose of this report was to describe myopic nontractional foveal detachment with features mainly associated with pachychoroid diseases.

## Methods

This retrospective study was performed at a single center according to the tenets of the Declaration of Helsinki. The study was prospectively approved by the Institutional Review Board of the Samsung Medical Center.

We retrospectively reviewed electronic medical records of Samsung Medical Center to identify those who had serous nontractional foveal detachment with high myopia between January 2010 and November 2018. high myopia was defined if refractive errors were − 6.0 diopters or more and/or the axial length was larger than 26.0 mm [9]. If the subjects had undergone refractive or cataract surgeries, then, their eyes manifesting obvious fundus changes related to pathologic myopia were also included. The eyes with history of ocular inflammation, history of vitreoretinal surgery, history of ocular trauma or glaucoma were excluded. On optical coherence tomography (OCT), the eyes were excluded if they had a macular or paramacular hole, vitreous or membrane traction on the fovea, or classic myopic CNV. Classic myopic CNV was diagnosed by the pattern on fluorescein angiography which showed well-defined hyperfluorescence in the early phases and leakage of fluorescein dye during the late phase. Also, subretinal hyper-reflective materials that represented by a highly reflective projection above retinal pigment epithelium was detected to signify type 2 CNV [4].

All subjects in the study had undergone fluorescein angiography (FA), indocyanine green angiography (ICGA) (Spectralis HRA + OCT; Heidelberg Engineering, Inc., Heidelberg, Germany), spectral domain-OCT (Spectralis HRA + OCT; Heidelberg Engineering, Inc., Heidelberg, Germany), OCT Angiography (OCTA) (DRI-OCT Triton, Topcon co., Tokyo, Japan). OCT imaging by raster scan was conducted for the existence of SRF, intraretinal fluid, retinal pigment epithelial layer, subfoveal choroidal thickness, choroidal vessel and macular hole to exclude. OCT images were raster scanned to exclude macular hole or traction. Subfoveal choroidal thickness was defined as the distance between the outer portion of retinal pigment epithelium below the foveal center to the inner surface of the choroidal–scleral junction. In this study, we utilized OCT scans to define pachyvessel as the large choroidal vessel occupying a significant portion of the choroid and manifesting as the attenuation of choriocapillaris and Sattler’s layer and dilated Haller’s layer beneath SRF [8]. On ICGA, choroidal vascular hyperpermeability was defined as the area of patch hyperfluorescence, and punctate hyperfluorescent spot was defined as pinpoint intense hyperfluorescent spot seen in late phase (over 10 min) [10]. The presence of choroidal vascular hyperpermeability and punctate hyperfluorescent spots was independently evaluated by two examiners (YKS, KHB), both of whom were masked to diagnosis and OCT results.

Eyes with nontractional foveal detachment in high myopia were categorized into two groups according to the presence of type 1 CNV. Group1 was defined as the presence of SRF without evidence of CNV on ICGA and OCTA. Localized serous detachments of the neurosensory retina with focal RPE detachment or diffuse RPE abnormality were found on OCT, but indistinct or diffuse leakage or nonspecific finding on FA and no evidence of CNV on multimodal imaging were noted in Group1. Interestingly, such features look similar to those observed in chronic CSC in myopic eyes. Herein, we used the term “myopic CSC” for such conditions. Group2 was defined as the presence of SRF with evidence of type 1 CNV on ICGA and/or OCTA. Localized serous detachment of neurosensory retina with focal RPE disruption or flat irregular RPE detachment (double-layer sign) were observed in Group2. There was evidence of CNV on multimodal imaging, but typical classic myopic CNV was not identified. Herein, we used the term “myopic pachychoroid neovascularization (PNV)” for such conditions.

Statistical analysis was executed using SAS version 9.4 (SAS Institute, Cary, NC) and R 3.5.1 (Vienna, Austria; http://www.R-project.org/). The best-corrected visual acuity was converted into logarithm of the minimal angle of resolution (LogMAR) units prior to the analysis. T-test and Wilcoxon rank sum test were conducted after the normality test respectively. *P*-values less than 0.05 were considered statistically significant.

## Results

A total of 15 eyes from 13 patients (6 men, 7 women) between January 2010 and November 2018 were included for analysis. Demographic and clinical characteristics of all patients were shown in Table 1. The mean age was 50.0 ± 10.5 years. The mean follow-up period was 37.3 ± 31.9 months (range, 1 to 99 months). Mean logMAR visual acuity at baseline and at the final visit was 0.22 ± 0.67 and 0.20 ± 0.60, respectively. Mean refractive error was − 7.8 ± 3.8 diopters and all eyes had the pathologic myopic feature on funduscopy. Three eyes had refractive surgery history, and 3 eyes had cataract surgery history at first visit. Only three eyes had axial length measurements for reasons of cataract history. The mean subfoveal choroidal thickness was 104.1 ± 27.4 μm.

**Table 1:** Demographic and clinical characteristics of all eyes

No.	Age	Sex	R/L	Follow up (Months)	Initial Snellen BCVA	Final Snellen BCVA	Refractive error	META-PM classification (chorioretinal atrophy)	Height of SRF at first visit (μm)	Treatment PDT/ Intravitreal anti-VEGF injection	Treatment response at final visit
Myopic CSC group											
1	43	F	R	11	20/50	20/32	−17	Patchy	15	0/6	Recurred
2	43	F	L	11	20/32	20/25	−14	Patchy	40	Untreated	Not changed
3	46	M	R	88	20/32	20/25	−5.5	Diffuse	37	Untreated	Disappeared
4	27	M	L	5	20/25	20/20	−6.5	Diffuse	48	1/9	Complete
5	67	F	R	93	20/100	20/63	−7.75	Diffuse	97	1/0	Complete
6	56	M	R	1	20/80	20/63	−4.75	Diffuse	112	1/0	Not available
7	40	F	L	7	20/25	20/32	−8	Diffuse	136	1/2	Incomplete
8	69	F	R	64	20/40	20/63	−6.25	Diffuse	67	Untreated	Not changed
Myopic PNV group											
9	69	F	L	64	20/40	20/63	−6	Diffuse	113	2/5	Incomplete
10	59	F	R	21	20/63	20/40	−3	Tessellated	102	0/5	Complete
11	59	M	R	99	20/63	20/100	−4.75	Diffuse	78	3/18	Incomplete
12	32	F	R	21	20/20	20/20	−9	Diffuse	61	1/1	Complete
13	46	F	L	7	20/25	20/32	−5	Diffuse	117	0/1	Refractory
14	46	M	L	12	20/32	20/25	−12.5	Diffuse	42	0/5	Incomplete
15	48	M	L	55	20/20	20/20	−9.5	Diffuse	35	0/2	Complete

*R/L* Right eye/Left eye, *CSC* Central serous chorioretinopathy, *PNV* Pachychoroid neovasculopathy, *BCVA* Best corrected visual acuity, *META-PM* META-analysis for Pathologic Myopia, *SRF* Subretinal fluid, *PDT* Photodynamic therapy, *VEGF* Vascular endothelial growth factor

Of the 15 eyes, 8 (53.3%) were in group1 (myopic CSC), and 7 eyes (46.7%) were in group2 (myopic PNV). The mean age was 48.9 ± 11.3 years for myopic CSC group and 51.1 ± 9.5 years for myopic PNV group. The logMAR visual acuities of myopic CSC and myopic PNV groups at baseline were 0.26 ± 0.76 and 0.18 ± 0.60, respectively. The logMAR visual acuities in both groups at the final visit were 0.21 ± 0.62 and 0.19 ± 0.58, respectively. Mean total follow-up period in myopic CSC and myopic PNV groups from baseline to the final visit was 35.0 ± 35.0 and 39.9 ± 28.1 months, respectively. Subfoveal choroidal thickness was 110.1 ± 29.4 μm in myopic CSC and 97.1 ± 25.3 μm in myopic PNV. There was no statistically significant difference. Five out of 8 eyes in myopic CSC group and all 7 eyes in myopic PNV group received treatment (Intravitreal anti-VEGF (vascular endothelial growth factor) injections and/or photodynamic therapy (PDT)). In the myopic PNV group, anti-VEGF injection was the first-line therapy. In the myopic CSC group, we applied half-fluence PDT as the first-line treatment in patients with foveal detachment accompanied by choroidal hyperpermeability on ICGA and who showed recent visual deterioration. In all other patients, we monitored the condition without intervention. At the final visit, Among the 11 eyes that underwent treatment, excluding one eye that was lost follow-up after treatment, were sorted according to their responses to treatment as followed: complete, incomplete, refractory and recurred response. Complete responses were noted in 5 eyes that 2 eyes from myopic CSC group, 3 eyes from myopic PNV group. Incomplete responses were noted in 4 eyes, 3 of which were in the myopic PNV group. Recurred response was noted in one eye in myopic CSC group, and refractory response was noted in one eye in myopic PNV group. Two of three untreated eyes in myopic CSC had no significant change and the last one had a spontaneous resolution of SRF (Tables 1, 2).

**Table 2:** Demographic and clinical characteristics of patients with to all eyes, myopic CSC and myopic PNV

	All eyes (*N* = 15) Mean ± SD	Myopic CSC (*N* = 8) Mean ± SD	Myopic PNV (*N* = 7) Mean ± SD	*P*-value
Age (years)	50.0 ± 10.5	48.9 ± 11.3	51.1 ± 9.6	0.73
Sex				1
Male/Female, n (%)	6 (40) / 9 (60)	3 (37.5) / 5 (62.5)	3 (42.9) / 4 (57.1)	
Follow-up period (months)	37.3 ± 31.9	35 ± 35	39.9 ± 28.1	0.38
Baseline BCVA, LogMAR	0.22 ± 0.67	0.26 ± 0.76	0.18 ± 0.60	0.52
Final BCVA, LogMAR	0.20 ± 0.60	0.21 ± 0.62	0.19 ± 0.58	0.97
Refractive error (Diopter)	−7.8 ± 3.8	−8.7 ± 4.5	−6.6 ± 3.2	0.49
SFCT (μm)	104.1 ± 27.4	110.1 ± 29.4	97.1 ± 25.3	0.51

*CSC* Central serous chorioretinopathy, *PNV* Pachychoroid neovascularization, *SD* Standard deviation, *BCVA* Best corrected visual acuity, *SFCT* Subfoveal choroidal thickness

Pachyvessels on OCT corresponding to the enlarged choroidal vessel on ICGA were noted in all eyes (8 eyes (100%) in myopic CSC and 6 eyes (100%) myopic PNV) in both groups. ICGA results were examined in all eyes in myopic PNV group except one eye (No.10). In myopic CSC group, 8 eyes (100%) showed choroidal vascular hyperpermeability and 5 eyes (62.5%), punctate hyperfluorescent spots. In the myopic PNV group, 5 eyes (83.3%) showed choroidal vascular hyperpermeability, and 5 eyes (83.3%) showed punctate hyperfluorescent spots. All multimodal imaging findings were reported in Table 3.

**Table 3:** Multimodal imaging findings of patients according to each group

Parameters	Myopic CSC (*N =* 8)	Myopic PNV (*N =* 7)
Funduscopy		
Tessellated fundus	0/8	1/7 (14.3%)
Diffuse atrophy	6/8 (75%)	6/7 (85.7%)
Patchy atrophy	2/8 (25%)	0/7
Macular atrophy	0/8	0/7
Staphyloma	2/8 (25%)	2/7 (28.6%)
OCT		
Pachyvessel beneath SRF	8/8 (100%)	7/7 (100%)
DSM	4/8 (50%)	2/7 (28.6%)
DLS	0/8	5/7 (71.4%)
IRC	0/8	2/7 (28.6%)
Subretinal HF	6/8 (75%)	4/7 (57.1%)
FA		
Leakage in early phase	0/8	1/7 (14.3%)
Stippled or diffuse leakage in late phase	0/8	6/7 (85.7%)
ICGA		
Choriodal vascular hyperpermeability	8/8 (100%)	5/6 (83.3%)
Punctate hyperfluorescent spot	5/8 (62.5%)	5/6 (83.3%)
OCTA		
Positive vascular density in outer retinal slab	0/6	5/5 (100%)

*OCT* Optical coherence tomography, *OCTA* Optical coherence tomography angiography, *FA* Fluorescence angiography, *ICGA* Indocyanine green angiography, *SRF* Subretinal fluid, *DSM* Dome-shaped macula, *DLS* Double layer sign, *IRC* Intraretinal cyst, *HF* hyper-reflective foci, *CSC* Central serous chorioretinopathy, *PNV*, Pachychoroid neovascularization

**Fig. 1:**
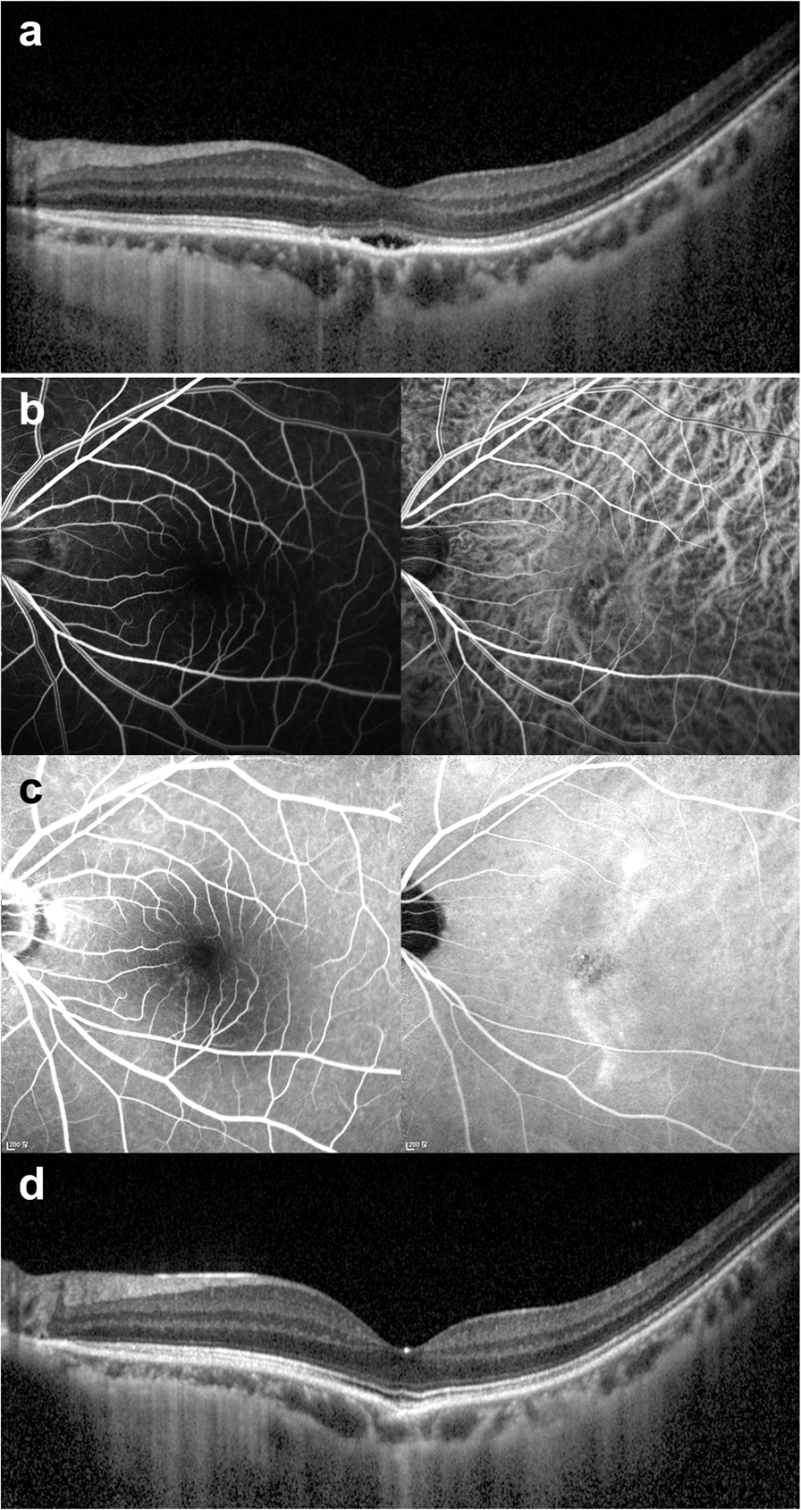
Representative myopic central serous chorioretinopathy (Case 4). A 20’s patient was referred to our clinic for the alleged diagnosis of central serous chorioretinopathy and had received previous 9 intravitreal anti-VEGF injections at another clinic. At presentation, refractive error was − 6.5 diopters, and best-corrected visual acuity was decreased to 20/25 in the left eye. A young patient complained visual discomfort. **a** Optical coherence tomography showed foveal detachment above focal choroidal thickening and the pachyvessel (arrowhead) and intrascleral vessel (arrow). There was no evidence of any vitreomacular traction or hole. **b** In the early phase, fluorescein angiography showed no definite leakage, and indocyanine green angiography showed hyperfluorescence overlying dilated vessel. **c** In the late phase, fluorescein angiography showed mild dye leakage, but there was no definite neovascularization, and indocyanine green angiography showed choroidal hyperpermeability. **d** On 13 months after photodynamic therapy, spectral domain optical coherence tomography showed complete resolution of subretinal fluid. Best-corrected visual acuity was improved to 20/20, and symptoms improved

**Fig. 2:**
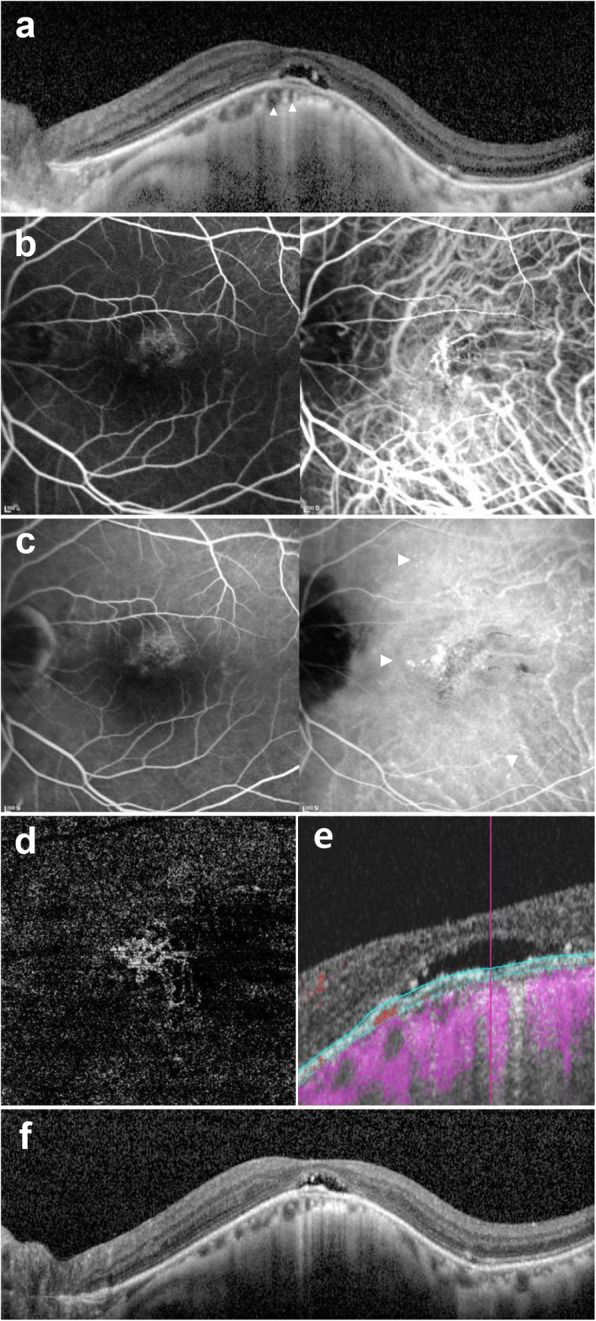
Representative myopic pachychoroid neovascularization (Case 9). A 60’s patient was referred to our clinic for evaluation of age-related macular degeneration. Patient did not have any previous medical history. At presentation, refractive error was − 6.0 diopters, and best-corrected visual acuity was decreased to 20/40 in the left eye. **a** Optical coherence tomography showed foveal detachment with double layer sign above pachyvessel (arrowhead). There was no evidence of any vitreomacular traction or hole. **b** In the early phase, fluorescein angiography showed ill-defined leakage, and indocyanine green angiography showed abnormal choroidal vessel and dilated choroidal vessel. **c** In the late phase, fluorescein angiography showed mild diffuse leakage similar to early phase and indocyanine green angiography showed choroidal hyperpermeability and punctate hyperfluorescence dot (arrowhead), choroidal leakage on choroidal neovascularization. **d** Outer-retinal slab on optical coherence tomographic agiography showed clear vascularity of choroidal neovascularization. **e** B scan flow overlay showed vascular flow (red color) within a double layer sign. **f** On 64 months after two photodynamic therapy and multiple anti-VEGF injection treatments under the diagnosis of type 1 choroidal neovascularization in myopia, optical coherence tomography showed persistent subretinal fluid. Best corrected visual acuity was slightly decreased to 20/63

Figures 1 and 2 show cases representing myopic CSC and myopic PNV.

## Discussion

Both high myopia and pachyspectrum diseases such as CSC and polypoidal choroidal vasculopathy are more common in the Asian population [9, 11]. However, pachychoroid diseases are very rare in high myopia. In this study, we describe high myopia which had nontractional foveal detachment with pachychoroid characteristics In this study, no eyes demonstrated intraocular inflammation.

There is a number of complications that require careful scrutiny when dealing with high myopic eyes exhibiting foveal detachment with SRF. Usually, these complications of high myopia include myopic CNV and myopic traction maculopathy. Myopic traction maculopathy is a wide spectrum of related disorders, encompassing vitreomacular traction, myopic foveoschisis and myopic macular hole. In severe forms of myopic traction maculopathy, OCT reveals foveal detachment and foveoschisis with posterior staphyloma in high myopia [12]. In the case of myopic CNV, FA and OCT are recommended for baseline diagnostic examination. In typical myopic CNV, FA reveals well-defined hyperfluorescence in early phases and leakage of fluorescein dye during the late phases, and OCT typically delineates a highly reflective area above the retinal pigment epithelium, namely subretinal hyper-reflective material, with minimal SRF. The relationship between dome-shaped macula and serous foveal detachment in eyes without typical myopic CNV was reported [13]. The morphologic changes in choroidal vascular structure were reported according to presence of posterior staphyloma [14]. However, the main focus of our report was to present serous non-tractional foveal detachment in highly myopic eyes with pachychoroid features and to classify them by presence of type 1 CNV.

Although FA and OCT are the most commonly conducted baseline examinations, in case of clear SRF in highly myopic eyes, these baseline examinations often fail to delineate myopic CNV [4]. ICGA may be more sensitive for detecting CNV or lacquer crack formation [15]. In 7 eyes of the myopic PNV group, the presence of the type1 CNV was confirmed via ICGA or OCTA.

In this study, all the eyes manifested myopic retinal changes consistent with the META-PM criteria [16]. However, all included eyes also showed common characteristics of pachychoroid features. Considering that retinal changes associated with pachychoroid spectrum disease stem from the structural instability inherent with the thick choroid, the naturally low prevalence of pachychoroid features in the thin choroid of high myopia is self-explanatory. In a previous study by Kim et al., choroidal vascular hyperpermeability was not noted in any of the eyes and punctate hyperfluorescent spot was noted in only 4 eyes (4.7%) among 84 eyes with classic myopic CNV [17]. Interestingly, the results in this study indicate that all myopic eyes with thin choroid, with or without type 1 CNV, shared pachychoroid phenotypes. On ICGA, choroidal vascular hyperpermeability, dilated choroidal vessel and punctate hyperfluorescent spot were detected in 13 (92.9%), 14 (100%) and 10 eyes (71.4%), respectively among the 14 eyes enrolled. This is also supported by previous reports claiming that together with dilated choroidal vessels, the punctate hyperfluorescent spot was a co-existing characteristic of choroidal vascular hyperpermeability [18]. On OCT, the presence of pachyvessels beneath SRF was noted in all 15 of 15 eyes (100%) of the current study. These results indicate that nontractional and non-rhegmatogenous serous foveal detachment in high myopia most likely accompany pachychoroid features.

FA and OCT are well-established tests for the diagnosis and monitoring of the activity of myopic CNV because myopic CNV is predominantly classic, type 2 CNV. Meanwhile, the detection of type 1 CNV within flat irregular RPE detachment most often requires on ICGA or OCTA examination. However, polypoidal choroidal vasculopathy and CSC have also been reported in highly myopic eyes [19, 20]. Five of 7 eyes in myopic PNV group had shallow irregular RPE detachment which appears as double layer sign, i.e. flat irregular RPE detachment overlying pachyvessel. The double layer sign indicates the presence of type 1 neovascularization, because CNV vascularity was detected in all of these eyes using ICGA and OCTA. Other 2 of 7 eyes in myopic PNV group showed slight hyper-reflective area with SRF above disrupted retinal pigment epithelium on OCT. Pachyvessels with the dilated choroidal vessel were also observed. This study is the first to address the presence of pachychoroid characteristics as the etiology of myopic serous foveal detachment.

Both ICGA and OCTA are very useful in studying choroidal vascularization especially in high myopia, and high sensitivity and specificity of OCTA are rendered useful in diagnosing myopic CNV [21]. Although the angiographic images in high myopia were difficult to interpret in many cases because of degenerative RPE changes, OCTA, nonetheless, provides high-resolution confocal images of type 1 CNV even in the presence of diffuse RPE window defect. In the current study, the high-flow network located in outer-retinal and choriocapillaris slab shown on OCTA was considered type 1 CNV in high myopia. Our results confirmed that OCTA is very valuable in analyzing outer-retinal neovascular structures for the eyes with serous foveal detachment in high myopia.

The intravitreal anti-VEGF injection is the first-line therapy for myopic CNV [22]. On the other hand, a combination of ranibizumab and PDT was superior to monotherapy for the treatment of non-myopic polypoidal choroidal vasculopathy in improving BCVA and achieving complete polyp regression [23]. In our study, all of 7 eyes in myopic PNV were treated with either intravitreal anti-VEGF injection or PDT, and 3 eyes achieved complete resolution and other 3 eyes, incomplete resolution. The refractory response was noted in one eye.

In myopic CSC group, half-fluence Verteporfin PDT was initially considered except for those with a history of anti-VEGF injection, and if there was an incomplete response, then anti-VEGF injection was performed. After PDT, complete resolution of SRF was noted in two of 3 eyes, while the incomplete response was noted in one eye. Some studies demonstrated PDT as a more favorable method than anti-VEGF injection in the treatment of atypical CSC [24]. In the present study, PDT may be considered as the first optional treatment in myopic CSC. However, further controlled study is required to establish the treatment principles in these groups of patients.

In the case of myopic PNV, our initial treatment was intravitreal anti-VEGF injection. If there was an incomplete resolution of SRF, we tried PDT in the area of choroidal hyperpermeability and SRF. After PDT, complete resolutions of SRF were noted in one of 3 eyes, while incomplete responses were noted in the other 2 eyes. Interestingly, these 2 eyes both had specific structural changes that involved the presence of staphyloma and dome-shaped macula. And incomplete response after PDT in myopic CSC was noted in one eye above that had a dome-shaped macula. According to previous studies, the primary cause of SRF development was not only choroidal vascular permeability, but also mechanical and vascular damage to the choroid by excessive scleral bulging [25]. Various structural changes accompanying high myopia may affect the treatment outcome and make treatment difficult. And these results suggested weaker response in the eyes with myopic PNV to anti-VEGF treatment when compared to anti-VEGF treatment for classic myopic CNV. Despite some time of investigation and clinical experiments since the initial report on PNV, the prognosis and treatment of non-myopic PNV with or without exudation has not been sufficiently addressed. We speculated that PNV is an arterialized mature neovascularization of type 1 CNV over a long period of slow vascular remodeling that increases tolerance and poor response to anti-VEGF treatment.

There were several limitations to the present study. First, the study design was a single-center, retrospective, short-follow up cases that could not represent the prevalence of overall population. Future studies looking at larger and longer series will be needed to characterize these conditions further and determine the optimal treatment approach. Second, the study population was small for both groups. However, this is because the nature of myopia and pachychoroid disease is difficult to coexist. Despite the limitations, this study has significance in that it elicited the interest in the clinical entity of myopic pachychoroid phenotypes as the etiology of myopic serous detachment. We provide new data on angiographic findings on OCTA and present our perspectives in diagnosing and treating myopic CSC and myopic PNV.

In conclusion, Pathologically myopic eyes could have pachychoroid features such as choroidal hyperpermeability or pachyvessel. In this study, we used ‘myopic CSC’ and ‘myopic PNV’ that demonstrate pachychoroid phenotypes. These may result in nontractional serous foveal detachment. In general, these are responsive to anti-VEGF treatment and/or PDT, but shows limited response. Despite of small series, our treatment experience broadens the perspective on disease entity overlapping between high myopia and pachychoroid spectrum disease.

## Data Availability

The data used to support the findings of this study are available from the corresponding author upon request.

## Abbreviations

CNV: Choroidal neovascularization
CSC: Central serous chorioretinopathy
RPE: Retinal pigment epithelium
SRF: Subreinal fluid
OCT: Optical coherence tomography
FA: Fluorescein angiography
ICGA: Indocyanine green angiography
OCTA: Optical coherence tomographic agiography
PNV: Pachychoroid neovascularization
LogMAR: Logarithm of the minimal angle of resolution
PDT: Photodynamic theraphy
VEGF: Vascular endothelial growth factor.
BCVA: Best corrected visual acuity
